# Wahrgenommene Einschränkungen während der COVID-19-Pandemie bei älteren Personen in der Schweiz

**DOI:** 10.1007/s16024-021-00364-w

**Published:** 2022-01-10

**Authors:** Alexander Seifert, Benedikt Hassler, Andreas Pfeuffer

**Affiliations:** Institut Integration und Partizipation, Hochschule für Soziale Arbeit der Fachhochschule Nordwestschweiz (FHNW), Riggenbachstr. 16, 4600 Olten, Schweiz

**Keywords:** Wohlbefinden, Einschränkungen, Corona, Bewegung, Sozialkontakte, Alter, Well-being, Restrictions, Coronavirus, Physical activity, Social contact, Aging

## Abstract

**Hintergrund:**

Die aktuelle Coronapandemie wirkt sich unterschiedlich auf das Alltagsleben älterer Menschen aus. Im Rahmen der pandemiebedingten Schutzmaßnahmen wurden insbesondere Personen ab 65 Jahren gebeten, direkte Kontakte und den Aufenthalt im öffentlichen Raum zu meiden.

**Ziel:**

Die vorliegende Arbeit untersucht, wie sich die Pandemie auf das Gefühl der eingeschränkten täglichen Versorgung, auf die Bewegung im Freien und die sozialen Kontakte bei Personen ab 50 Jahren ausgewirkt hat.

**Material und Methode:**

Zwischen Mai und Juni 2020 wurden 1011 in der Schweiz lebende Personen ab 50 Jahren telefonisch befragt. Das Durchschnittsalter der Befragten liegt bei 65 Jahren, und 53 % der befragten Personen sind Frauen.

**Ergebnisse:**

Die Untersuchung zeigt, dass die befragten Personen insgesamt kaum negative Veränderungen in Bezug auf ihre Versorgung mit Dingen des alltäglichen Gebrauchs oder Bewegung im Freien spürten. Jedoch gaben 43 % der Befragten an, während der Pandemie und den damit verbundenen Schutzmaßnahmen häufiger das Gefühl gehabt zu haben, zu wenig Zeit mit Menschen verbringen zu können, die ihnen wichtig sind. Multivariate Auswertungen zeigen, dass bei der Bewertung der drei Alltagsbereiche der Faktor Bildung eine Rolle spielt.

**Schlussfolgerung:**

Die Studie zeigt mögliche Alltagseinschränkungen älterer Menschen unter Pandemiebedingungen auf und sollte zur Diskussion anregen, um die subjektiven Wahrnehmungen der älteren Menschen in der praktischen gerontologischen Arbeit besser berücksichtigen zu können.

## Abstract (en)

**Background:**

The current coronavirus pandemic has had various effects on older people’s everyday lives. Within the framework of pandemic-related protective measures, people over 65 years of age in particular were asked to stay away from public places and avoid direct physical contact.

**Aim:**

This paper examines how the pandemic affected the feelings of people over 50 years of age with regard to the limited daily supply of everyday things, outdoor physical activity and social contact.

**Material and methods:**

In May and June 2020, telephone interviews were conducted with 1011 people aged 50 years and over living in Switzerland. The average respondent age was 65 years and 53% of the respondents were women.

**Results:**

The results show that the respondents hardly felt any negative changes in their supply of everyday things or outdoor activity; however, 43% of those surveyed stated that during the pandemic, with its associated protective measures, they more often felt that they were unable to spend enough time with people they cared about. The multivariate results show that education played a role in the respondents’ evaluation of everyday life considered in the interviews.

**Conclusion:**

This study identifies older people’s feelings about possible everyday restrictions and should stimulate discussion in practical gerontological work to better consider older people’s subjective perceptions.

## Hintergrund

Weltweit ist der Alltag der meisten Menschen derzeit von der Coronapandemie (COVID-19-Pandemie) geprägt (Ayalon et al. 2020; Brooke und Jackson 2020). Seit Beginn der Pandemie sind insbesondere ältere Menschen, genauer gesagt Personen ab 65 Jahren, in Privathaushalten und stationären Alterspflegeeinrichtungen als „besonders gefährdete Personen“ deklariert worden; ihnen wurde nahegelegt, sich zu Hause zu isolieren und nach Möglichkeit auf soziale Kontakte zu verzichten (Bundesamt für Gesundheit 2020). Ältere Menschen finden sich während der Pandemie in einem Alltag wieder, der durch mögliche Einschränkungen z. B. in der Versorgung mit Dingen des alltäglichen Gebrauchs, der Bewegung im Freien und der Möglichkeiten, Zeit mit Menschen, die ihnen wichtig sind, verbringen zu können, gekennzeichnet ist. Diesen drei Themen (Versorgung, Bewegung, soziale Kontakte) widmete die vorliegende Untersuchung besondere Beachtung, da sie alltägliche und für das psychische wie physische Wohlergehen grundlegende Lebensbereiche älterer Menschen darstellen.

Die andauernde Pandemie kann demzufolge als „Stresssituation“ angesehen werden, da direkte soziale Kontakte oder alltägliche Dinge wie das Einkaufengehen seltener stattfinden (Polizzi et al. 2020). Wie aber erleben ältere Menschen die Alltagseinbußen während der Pandemie und wie reagieren sie auf diese? Verlässliche empirische Daten, wie sich die Pandemie auf die subjektive Wahrnehmung von Veränderungen bei der täglichen Versorgung, den Bewegungsmöglichkeiten und der Zufriedenheit mit den sozialen Kontakten bei älteren Menschen auswirkt, ließen sich zum Zeitpunkt der Beitragserstellung kaum finden.

Bislang konnte gezeigt werden, dass das Gefühl von Einsamkeit in der Schweizer Bevölkerung ab 65 Jahren mit Beginn der Schutzmaßnahmen und der damit verbundenen Empfehlung (Bundesamt für Gesundheit 2020) an Personen ab 65 Jahren, nach Möglichkeit zu Hause zu bleiben, zunächst leicht angestiegen ist und sich später wieder normalisiert hat (Höglinger et al. 2020; Seifert und Hassler 2020). Außerdem zeigen erste Ergebnisse, dass sich einige Personen seit Beginn der Pandemie weniger sportlich betätigen und sich auch seltener im Freien aufhalten. Diese „Inaktivität“ wirkt sich negativ auf deren Wohlbefinden aus (Lesser und Nienhuis 2020). Dennoch ist die Forschungslage – gerade bezüglich der älteren Bevölkerungsgruppe – eher dürftig, und es bleibt die Frage offen, wie diese Menschen die Veränderungen (hier am Beispiel der Bereiche Versorgung, Bewegung, soziale Kontakte) konkret wahrnehmen und bewerten.

## Forschungsfrage und Ziele

Der vorliegende Beitrag verfolgt das Ziel, anhand empirischer Daten Aussagen darüber zu treffen, inwieweit ältere Personen ab 50 Jahren Veränderungen für ihr tägliches Leben bilanzierend (in diesem Sinne retrospektiv) wahrnehmen – also im Vergleich von „vor der Pandemie“ und „mit Beginn der Pandemie“. Hierbei werden verschiedene Lebensbereiche betrachtet: a) Versorgung mit Dingen des täglichen Gebrauchs, b) Bewegungsmöglichkeit im Freien und c) Zufriedenheit mit den sozialen Kontaktmöglichkeiten. Zudem wird herausgearbeitet, wer von den Befragten insbesondere negative Veränderungen wahrnimmt, um so auf soziale Ungleichheiten innerhalb dieser Altersgruppe schließen zu können.

Hieraus ergeben sich zwei Forschungsfragen:

1. Wie werden die Versorgung mit Dingen des täglichen Gebrauchs, die Bewegungsmöglichkeit im Freien und die Zufriedenheit mit den sozialen Kontaktmöglichkeiten im Vergleich zur Situation vor der Pandemie eingeschätzt?
2. Welche personenbezogenen (Alter, Geschlecht, Bildung, Einkommen, Kinder, Kontakthäufigkeit zu Freunden) und kontextbezogenen (Wohnform und Wohnort) Faktoren beeinflussen diese Einschätzungen?

## Methode

### Vorgehen

Um herauszufinden, wie Personen ab 50 Jahren die Veränderungen ihres Alltags während der COVID-19-Pandemie beurteilen, wurden Fragen in eine Omnibusbefragung des Befragungsinstituts gfs-zürich eingepflegt. Die Befragung erfolgte in der Zeit vom 19.05.2020 bis zum 16.06.2020. Telefonisch („computer assisted telephone interview“) wurden insgesamt 1011 Personen ab 50 Jahren aus der deutsch- (*n* = 761) bzw. französischsprachigen Schweiz (*n* = 250) befragt, wobei die AZ-Direct-Adressbasis (basierend auf dem Telefonbuch) genutzt wurde. Die italienischsprachige Schweiz wurde in der Befragung nicht berücksichtigt. Die Rücklaufquote lag bei 12,2 %.

### Variablen und statistische Auswertung

Im Rahmen der Befragung sollten die möglichen Veränderungen des Alltags bilanzierend bewertet werden: „Bitte sagen Sie mir, wie sich der jeweilige Aspekt in Ihrem Leben seit Beginn der Coronapandemie (COVID-19; seit den Corona-bedingten Einschränkungen in der Schweiz) für Sie verändert hat.“ Die Antworten wurden mittels einer elfstufigen Skala erhoben, die von (−5) „es hat sich stark negativ verändert“ über (0) „weder … noch“ bis (+5) „es hat sich stark positiv verändert“ reichte. Erfragt wurden folgende Aspekte: a) „meine Versorgung mit Dingen des täglichen Lebens“ (z. B. Lebensmittel, Hygieneartikel), b) „meine Bewegungsmöglichkeit an der frischen Luft (im Freien, außer Haus)“, c) „mein Gefühl, dass ich genügend Zeit mit Menschen verbringen kann, die mir wichtig sind“. Als Kontrollvariablen fungierten die in Tab. 1 aufgeführten Variablen. Es wurden deskriptive Beschreibungen erstellt und multivariate lineare Regressionen gerechnet, um die Einflussfaktoren auf die wahrgenommenen Veränderungen herauszustellen. Die Berechnungen erfolgten mit SPSS (IBM, Armonk, NY, USA) Version 26.0.

**Table 1:** 

Merkmale		Anzahl	Gültige Prozente in Stichprobe
Stichprobe Gesamt		1011	100,0
Geschlecht	Frau	532	52,6
	Mann	479	47,4
	*Weiß nicht/keine Angabe*	–	–
Altersgruppen	50–59 Jahre	387	38,3
	60–69 Jahre	278	27,5
	70–79 Jahre	231	22,8
	80–89 Jahre	109	10,8
	90+	6	0,6
	*Weiß nicht/keine Angabe*	–	–
Bildung	Obligatorische Schule	107	10,7
	Sekundarstufe II (Berufsbildung)	299	30,0
	Sekundarstufe II (Allg.-Bildung)	65	6,5
	Tertiärstufe (höhere Berufsbild.)	149	14,9
	Tertiärstufe (Hochschulen)	377	37,8
	*Weiß nicht/keine Angabe*	14	–
Haushaltseinkommen	Bis 4000	152	17,2
	4001–6000	224	25,4
	6001–9000	230	26,1
	Mehr als 9000	276	31,3
	*Weiß nicht/keine Angabe*	129	–
Siedlungstyp	Stadt	315	31,2
	Agglomeration	414	40,9
	Land	282	27,9
	*Weiß nicht/keine Angabe*	–	–
Alleinlebend	Allein wohnend	287	28,4
	Nicht allein wohnend	722	71,6
	*Weiß nicht/keine Angabe*	2	–
Haushaltsform	Zur Miete	351	35,0
	Eigentum	653	65,0
	*Weiß nicht/keine Angabe*	7	–
Kinder vorhanden und Kontakt zu Kindern	Ja	774	76,9
	Nein	232	23,1
	*Weiß nicht/keine Angabe*	5	–
Kontakt zu Freunden	Täglich	109	11,0
	Mehrmals in der Woche	355	35,9
	Einmal in der Woche	236	23,9
	Mehrmals im Monat	154	15,6
	Seltener	101	10,2
	Nie	33	3,3
	*Keine Angabe*	23	–

## Ergebnisse

### Realisierte Stichprobe

An der Befragung haben 1011 Personen ab 50 Jahren teilgenommen. Merkmale der realisierten Stichprobe können der Tab. 1 entnommen werden. 52,6 % der befragten Personen sind Frauen. Die Altersspanne in der Stichprobe reicht von 50 Jahren (aufgrund der methodischen Festlegung) bis zu 95 Jahren. Im Durchschnitt sind die befragten Personen 65,4 Jahre alt (SD: 10,24). 25,4 % der Befragten verfügen über ein monatliches Haushaltsnettoeinkommen, das zwischen 4000 und 6000 Franken liegt. 17,2 % haben ein tieferes und 57,4 % ein höheres Einkommen. 40,9 % der Befragten wohnen in der Agglomeration (suburbanes Umland), 31,2 % in der Stadt und 27,9 % auf dem Land. Die überwiegende Mehrheit der Personen lebt gemeinsam mit anderen Personen in einem Haushalt (71,6 %), 65,0 % wohnen in ihrem eigenen Haus oder in einer Eigentumswohnung, und 34,9 % leben in einer Mietwohnung. Von den befragten Personen gaben 23,1 % an, keine Kinder bzw. keinen Kontakt zu ihren Kindern zu haben. Vor der Pandemie hatten 46,9 % der befragten Personen täglich oder mehrmals in der Woche Kontakt zu Freunden (Tab. 1).

### Versorgung, Bewegung und soziale Kontakte

**Figure 1:**
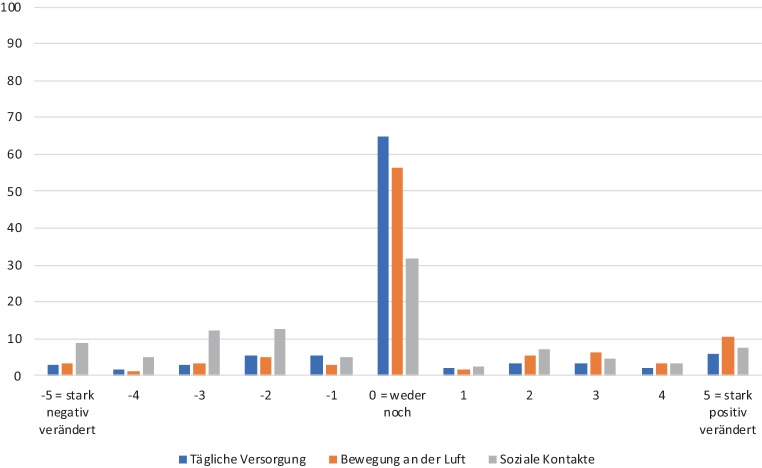

Auf der verwendeten Skala von (−5) „es hat sich stark negativ verändert“ bis (+5) „es hat sich stark positiv verändert“ ergibt sich für die Versorgung mit den täglichen Dingen ein Mittelwert von 0,08 (SD: 1,95). Dies bedeutet, dass die Befragten im Durchschnitt meinen, dass sich die Möglichkeit, eine tägliche Versorgung zu gewährleisten, während der Pandemie nicht wesentlich verändert hat. 18,6 % der Befragten erklärten, dass sich diese Versorgung für sie negativ verändert habe (Abb. 1).

Für die Möglichkeit, sich an der frischen Luft zu bewegen, ergibt sich ein Mittelwert von 0,53 (SD: 2,31). Dies bedeutet, dass die Befragten im Durchschnitt meinen, dass sich die Möglichkeit der Außerhausbewegung während der Pandemie kaum verändert hat. Nur 16,1 % vertraten die Einschätzung, dass sich ihre Bewegungsmöglichkeit negativ verändert hat (Abb. 1).

Mit der Aussage „Mein Gefühl, dass ich genügend Zeit mit Menschen verbringen kann, die mir wichtig sind“ konnte zusätzlich erfasst werden, wie zufrieden die Befragten mit der Möglichkeit der Wahrnehmung von sozialen Kontakten während der Pandemie im Vergleich zur Situation „vor Corona“ sind. Hier ergab sich ein Mittelwert von −0,47 (SD: 2,74), und 43,1 % gaben an, dass sie seit Pandemiebeginn stärker das Gefühl haben, zu wenig Zeit mit ihnen wichtigen Personen verbringen zu können. Hinsichtlich des Alters der befragten Personen und des Geschlechts ergeben sich bivariat keine statistisch signifikanten Unterschiede bei allen drei Bereichen der Alltagsveränderungen (Versorgung, Bewegung, Kontakte).

### Faktoren, welche die Wahrnehmung der Veränderungen beeinflussen

Um herauszufinden, wie die Soziodemografie, die Wohnsituation und die generelle Kontakthäufigkeit zu Freunden die Bewertungen der Alltagsveränderungen beeinflussen, wurden multivariate lineare Regressionen berechnet (Tab. 2). Abhängige Variable im ersten Modell ist die Versorgung, im Modell 2 sind es die Bewegungsmöglichkeiten und im Modell 3 die sozialen Kontakte.

**Table 2:** 

Prädiktoren	Modell 1: Tägliche Versorgung	Modell 2: Bewegung an der Luft	Modell 3: Soziale Kontakte
	Gesamt	Gesamt	Gesamt
Alter (50–95 Jahre)	0,005	0,073*	0,008
Frau (ref. Mann)	−0,042	−0,021	−0,052
Bildung (Skala 1–5)	−0,108**	−0,113**	−0,112**
Einkommen (Skala 1–4)	−0,026	−0,017	−0,039
Alleinlebend (ref. nicht alleinlebend)	−0,012	−0,121**	−0,049
Eigentum (ref. zur Miete)	−0,017	0,071	0,024
Ländlich (ref. nicht ländlich)	−0,016	0,024	0,034
Kinder bzw. Kontakt zu Kindern vorhanden (ref. nicht vorhanden)	−0,002	0,014	−0,009
Kontakthäufigkeit zu Freunden (Skala 1–6)	0,078*	0,087*	0,036
*Korrigiertes R*^*2*^	0,008*	0,032***	0,010*

Variablenskalen: Tab. 1. Lineare Regression (Methode: Eingabe)

*ref* Referenz

Signifikanzniveaus: ***p < 0,001, **p < 0,01, *p < 0,05

Im ersten Modell (tägliche Versorgung) haben nur die Bildung und die Kontakthäufigkeit zu Freunden einen statistisch signifikanten Einfluss auf die Wahrnehmung der Einschränkungen. Personen mit höherer Bildung und Personen, die seltener Kontakt zu Freunden haben, gaben stärkere Einschnitte in der Versorgung an als Personen mit niedrigerer Bildung sowie Personen, die häufiger Kontakt zu Freunden haben. Im zweiten Modell (Bewegung an der frischen Luft) sind das Alter, die Bildung, das Alleinleben sowie die Kontakthäufigkeit zu Freunden statistisch signifikant. Jüngere Personen, Personen mit höherer Bildung, alleinlebende Personen sowie Personen, die selten Kontakt zu ihren Freunden haben, berichteten diesbezüglich über stärkere Einschnitte. Im dritten Modell (soziale Kontakte) trägt nur die Bildung statistisch signifikant zur Erklärung bei. Personen mit einer höheren Bildung nehmen mehr negative Einschränkungen wahr als Personen mit einem niedrigeren Bildungsstatus.

## Diskussion

Mit der vorliegenden Studie konnten Informationen zur Wahrnehmung von Veränderungen im Alltagsverhalten bei der Schweizer Bevölkerung ab 50 Jahren erhoben werden. Die Studie ermöglicht einen deskriptiven Einblick in die Folgen der COVID-19-Pandemie für ältere Menschen, die von der Schweizer Regierung während der Pandemie als „besonders schützenswert“ deklariert wurden (Bundesamt für Gesundheit 2020). In Bezug auf die erste Forschungsfrage zeigte die Untersuchung auf, dass die befragten Personen insgesamt kaum negative Veränderungen bei der Versorgung mit täglichen Dingen oder der Bewegungsfreiheit bzw. Bewegung an der frischen Luft empfanden. Jedoch gaben einige Personen an, während der Pandemie und der damit verbundenen Schutzmaßnahmen häufiger das Gefühl gehabt zu haben, zu wenig Zeit mit Menschen verbringen zu können, die ihnen wichtig sind.

Mit der zweiten Forschungsfrage sollte erhoben werden, welche Faktoren die Wahrnehmung von negativen bzw. positiven Veränderungen beeinflussen. Über alle Modelle hinweg zeigten sich nur wenige unabhängige Variablen als statistisch signifikant; auch waren die jeweiligen erklärten Varianzen gering, was darauf hindeutet, dass noch andere Faktoren für die Wahrnehmungen der befragten Personen verantwortlich sein müssen. Dennoch konnten gewisse Ungleichheiten in der persönlichen und kontextuellen Situation der Befragten nachgewiesen werden. So zeigten sich bei allen drei Modellen Unterschiede hinsichtlich des Aspekts „Bildung“: Personen mit höheren Bildungsabschlüssen sprachen häufiger von Einschnitten in ihren Versorgungs‑, Bewegungs- und Kontaktmöglichkeiten als Personen mit niedrigeren Bildungsabschlüssen. Dies macht deutlich, dass Bildungsdifferenzen möglicherweise die Wahrnehmung von Einschränkungen beeinflussen können. So könnte z. B. die Bildung Einfluss darauf haben, inwieweit die staatlichen Vorgaben zur Einhaltung der Schutzmaßnahmen eingehalten werden. Erste Forschungsergebnisse weisen darauf hin (Georgiou et al. 2020), jedoch bedarf es hier weiterer Studien.

Bei der Versorgung mit Dingen des täglichen Bedarfs wirkte sich außerdem die Kontakthäufigkeit zu Freunden positiv auf die Wahrnehmung der Einschnitte aus. Dies könnte bedeuten, dass sich „Hilfsnetzwerke“ zwischen Freunden etabliert haben und somit eine Versorgung unter einer möglichen Quarantäne gewährleistet wurde. Bei dem Modell zur Bewegungsmöglichkeit an der frischen Luft wirkten neben der Bildung auch das Alter, das Alleinleben und die Kontakthäufigkeit mit Freunden. Hinsichtlich des Alters könnte es sein, dass sich ältere Personen bereits vor der Pandemie weniger außer Haus bewegt hatten und dadurch auch nach Beginn der pandemiebedingten Restriktionen weniger Einschränkungen erlebten. Ob dies tatsächlich so ist, kann mit den vorliegenden Daten nicht abschließend beantwortet werden. Hinsichtlich der Wohnsituation zeigt die Befragung auf, dass insbesondere Personen, die allein leben, negative Einschnitte erlebt haben – dies vielleicht auch, weil sie weniger soziale Motivation bzw. soziale Sicherheit durch eine Partnerin bzw. einen Partner hatten, sich außerhalb der eigenen vier Wände zu bewegen. Vergleichbares zeigt sich auch bei den Kontakthäufigkeiten zu Freunden, sodass Personen mit vielen Kontakten hier auch weniger negative Veränderungen wahrnehmen.

Auch wenn die Untersuchung keine „deutlich negativen Effekte“ – wie vielleicht vermutet – der Coronapandemie auf die Versorgung mit den Dingen des täglichen Bedarfs und die Bewegungsfreiheit an der frischen Luft nachweisen konnte, wurden jedoch Einschnitte dahingehend festgestellt, weniger Zeit mit emotional wichtigen Personen verbringen zu können. Dieses Gefühl, „zu wenig Zeit mit wichtigen Menschen verbringen zu können“, kann evtl. auch das Gefühl der Einsamkeit und sozialen Isolation vergrößern (Armitage und Nellums 2020; Losada-Baltar et al. 2020); eine Annahme, die anhand der vorliegenden Daten jedoch nicht abschließend beantwortet werden kann.

Dennoch sollten aber auch jene Personen (etwa 16–19 %) innerhalb der praktischen Gerontologie nicht vernachlässigt werden, die in Bezug auf die Versorgung und Bewegungsfreiheit von negativen Einschnitten gesprochen haben. Hier kann allenfalls darüber diskutiert werden, ob sich die von der Schweizer Regierung als „besonders gefährdete Gruppe“ definierten älteren Personen nicht gerade durch diese Zuschreibung zusätzlich „sozial isoliert“ und ausgegrenzt fühlten – schließlich sollte der direkte Kontakt zu ihnen von einem auf den anderen Tag vermieden werden. Wenn alle älteren Menschen pauschal als alt, krank, fragil und hilflos stigmatisiert werden, wird ein Altersbild bestärkt, das vielen in der Altersarbeit und Altersforschung tätigen Personen Sorgen bereitet, weil es die Altersdiskriminierung fördert und die Beziehungen zwischen Jung und Alt belastet (Ayalon et al. 2020) – und dies in einer ohnehin angespannten Zeit.

### Schlussfolgerung

Die vorliegende Untersuchung macht deutlich, dass ältere Personen während der COVID-19-Pandemie nur z. T. negative Einschnitte in der tagtäglichen Versorgung und Bewegung außer Haus erleben, was bis anhin vermutet wurde. Allerdings zeigt die Untersuchung auf, dass ein bedeutender Teil (43 %) der hier befragten Gruppe stärker als vor der Pandemie das Gefühl hat, nicht mehr genügend Zeit mit liebgewonnenen Menschen verbringen zu können. Aber auch die etwa 16–18 % jener Personen, die von Einbußen in Bezug auf die Versorgung und Bewegung im Freien sprachen, sollten nicht als „marginale Gruppe“ betrachtet und vernachlässigt werden. Vielmehr sollte hier gefragt werden, wie die praktische Gerontologie auf die Probleme jener Menschen reagieren kann, die sich durch die Kontakteinschränkungen sozial isoliert fühlen. Soziale Innovationen sind daher gefragt, die für allfällig auftretende vergleichbare Situationen Formen und Orte der Betreuung für ältere Menschen schaffen, die deren Anspruch auf Schutz, aber auch auf Selbstbestimmung und die Teilhabe am sozialen Leben wahren.

### Limitationen

Als Limitation ist zum einen anzugeben, dass nur Personen befragt wurden, die in Privathaushalten leben, Personen, die in stationären Alterseinrichtungen wohnen, wurden also nicht berücksichtigt; jedoch wäre auch ihre Einschätzung sehr interessant, da sie noch stärker von Besuchsverboten während der Pandemie betroffen waren. Es besteht zudem die Gefahr, dass durch die Form der telefonischen Befragung bestimmte Personengruppen (wie z. B. Personen mit Hörbeeinträchtigungen) systematisch aus dem Sample ausgeschlossen wurden. Zum anderen wurde innerhalb der Befragung nur bilanzierend bzw. retrospektiv gefragt; es wäre aber sehr wichtig, Daten zukünftig vermehrt längsschnittlich zu sammeln, um auch Veränderungen bei den Einzelpersonen im Zeitverlauf erfassen zu können. Darüber hinaus wäre es aufschlussreich, in zukünftigen Studien auch Fragen zum COVID-19-Befund, zur allgemeinen Krankheits‑, Betreuungs- und Pflegesituation, zum sozialen Netzwerk der älteren Menschen und zu den Bewertungen der Einschränkungen durch die Coronaschutzmaßnahmen aufzunehmen. Schließlich wäre es von großem Interesse, in zukünftigen Studien die subjektive Bewertung der erlebten Einschränkungen auch mittels qualitativer Erhebungsmethoden zu erfassen, um so die individuellen Lebenskontexte zu berücksichtigen.
